# Effect of retraction materials on gingival health: A histopathological study

**DOI:** 10.4103/0972-124X.65436

**Published:** 2010

**Authors:** Sushma Phatale, P.P. Marawar, Girish Byakod, Sanjay B. Lagdive, Jitendra V. Kalburge

**Affiliations:** *Department of Periodontics and Oral Implantology, Pravara Institute of Medical Sciences, Rural Dental College, Ahmednagar, Maharashtra, India*; 1 *Department of Prosthodontics, Pravara Institute of Medical Sciences, Rural Dental College, Ahmednagar, Maharashtra, India*; 2 *Department of Pathology, Pravara Institute of Medical Sciences, Rural Dental College, Ahmednagar, Maharashtra, India*

**Keywords:** Expasyl, magic foam cord, junctional epithelium, retraction cord

## Abstract

**Background:**

Gingival retraction methods are used in dentistry for impressions of subgingival crown margins, such as, mechanical, chemical, chemicomechanical, and surgical. These methods may injure the gingival sulcular epithelium. Hence, the present study is carried out to evaluate the effect of different retraction materials, such as, Expasyl, Magic Foam Cord, and impregnated retraction cord on the gingival sulcular epithelium.

**Materials and Methods:**

This study included 30 cases of bilateral premolar extraction patients with Loe and Silness gingival index zero. Retraction materials were kept in the dry, isolated labial gingival sulcus for the required time. The retraction materials were removed by rinsing with water. Retracted gingiva of 2 – 3 mm from the gingival margin along with the tooth was extracted and the decalcified sections were microscopically studied. Data analysis: Data were analyzed by applying the chi-square test.

**Results:**

This study showed better results with retraction paste as compared to the retraction cord, and there was a significant association between retraction materials and the relative degree of injury to the sulcular epithelium.

**Conclusion:**

There is a significant association between retraction materials and gingival sulcular epithelium. It can be stated that impregnated retraction cord, may be used commonly but it needs proper tissue manipulation and is technique sensitive. Newly advanced material in the form of retraction paste like Expasyl or Magic Foam Cord was found to be better than cord as assessed histologically, it respects periodontium.

## INTRODUCTION

Impressions for subgingival crown margins require gingival tissue retraction. Conservative retraction methods involving tissue displacement include the placement of copper bands or cords with or without caustics and astringents. In other methods, the gingival tissue is excised, as in resection by electrosurgery. Copper-band impression was indicated as the major factor producing gingival recession. Also sulcus damage with electrosurgery was reported to vary depending on the type of unit used.[1]

The relationship between periodontal health and restoration of teeth is intimate and inseparable. For restoration to survive long term, the periodontium must remain healthy so the teeth are maintained. For the periodontium to remain healthy, restoration must be critically managed in several areas so that they are in harmony with the surrounding periodontal tissue. Restorations play an important role in the ecological balance of plaque and maintenance of the periodontium.[2] If a margin of restoration has to be placed supragingivally or equigingivally then there is no need for gingival retraction. However, in unavoidable conditions, like in anterior restorations, for esthetic purposes, margins must be placed subgingivally; hence, it needs gingival retraction procedures, which may cause a violation of biological width. The dimension of the space that the healthy gingival tissue occupies above the alveolar bone is called the ‘biologic width’. This comprises of 1.07 mm of connective tissue attachment and 0.97 mm of junctional epithelium. The biologic width should not be violated in any restorative procedure. The average biological width is 2.04 mm.[3]

Various gingival retraction methods are mechanical, mechanochemical, electrosurgery, rotary gingival curettage, etc. The most commonly used method is the mechanochemical one. Use of the mechanochemical method leads to violation of biological width, causing bone loss and recession. Studies on the chemicomechanical and purely mechanical cord retraction techniques have shown various degrees of necrosis and/or stripping of the gingival sulcus.[4] Gingival electrosurgery for crevicular troughing involves a considerable risk of producing permanent periodontal damage.[5]

Very few histological studies have been reported on the effects of using retraction materials on the gingival sulcular tissue, although disruption of the sulcular epithelium could be expected. Hence, this study has been carried out to identify whether chemicomechanical and mechanical retraction materials injure the gingival sulcus epithelium. If so, which retraction material is better and causes less injury.

### Aims and objectives

Dental surgeons generally believe that retraction materials do not cause injury to the gingival sulcus epithelium. As injury to sulcular epithelium cannot be detected clinically, except after the most severe damage, this study is based on histological findings.

1. To determine the effect of the most commonly used retraction materials: Expasyl, Magic Foam Cord, and impregnated retraction cord on gingival sulcular epithelium.
2. To find out the association between Expasyl, Magic Foam Cord, and the impregnated retraction cord and gingival sulcular epithelium.

## MATERIALS AND METHODS

Thirty patients of age 11–17 years, with bilateral first premolar extraction cases in both maxillary and mandibular arches, were selected, irrespective of sex, who referred to the Orthodontia Department Rural Dental College, Loni, with Loe and Silness gingival index zero. Patients with improper oral hygiene, crowding, bleeding on probing, periodontal pocket, gingival recession or enlargement and any systemic diseases or conditions were excluded from the study. The protocol was clearly explained to all the patients and informed consent was obtained from all the recruits.

### Material

A) Retraction paste

- Expasyl — Aluminum chloride (15%), Kaolin, Water (Satelec ACTEON group)
- Magic Foam Cord — Polyvinylsiloxane, addition type silicone elastomer

vailable in form of Base — White; Catalyst — Blue (Coltene / Whaledent AG, Switzerland)

B) Retraction cord

Impregnated retraction cord with 5% Aluminum Chloride (Ultrapak, Ultradent products, Inc., Germany)

### Retraction procedure for Expasyl

Expasyl is a paste for temporary gingival retraction that ensures separation of the marginal gingiva and drying of the sulcus. The material is supplied in capsules (cartridges), and comes with a preformed gun-type of device into which the capsule has to be placed and then the material is expressed. Labial gingival sulcus of the maxillary right first premolar was rinsed with water, dried with air stream and isolated with cotton rolls. The retraction paste was slowly injected into the sulcus (2 mm/s) with the tip parallel to the long axis of the teeth, as shown in Figure 1a. The point of the cannula must create a closed space between the tooth and the marginal edge of the gingiva. Clinically, the complete filling of the sulcus can be discerned by a slight blanching of the gingival marginal area.[6] Depending on the tonicity of the gingiva it is kept in place for one minute in the thin and two minutes in the thick marginal gingiva. It is easily visible because of its color. Subsequently, it is removed by air and water spray.

**Figure 1a:**
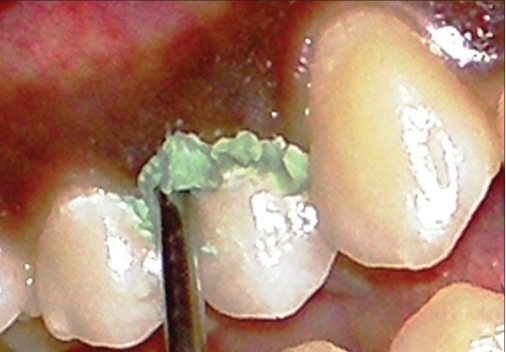
Expasyl paste in gingival sulcus of maxillary right 1^st^ premolar

### Retraction procedure for Magic Foam Cord

As shown in Figure 1b, the labial gingival sulcus of the mandibular left first premolar was rinsed with water and dried with an air stream. A Magic Foam Cord cartridge was placed in the dispenser and the cartridge cap removed. The handle was compressed to express some material onto a paper until the base and catalyst flowed out of the opening in equal amounts, which ensured an optimum mixture. The oral tip was placed onto the mixing tip. The Magic Foam Cord was slowly injected into the sulcus and then the Comprecap Anatomic was placed. Due to the counter pressure of the Comprecap Anatomic, there was an expansion of the Magic Foam Cord in the sulcus. It was kept in place for five minutes. Subsequently, after proper setting, both the Magic Foam Cord and Comprecap were removed in one piece. Next the Magic Foam Cord was completely removed by air and water spray.

**Figure 1b:**
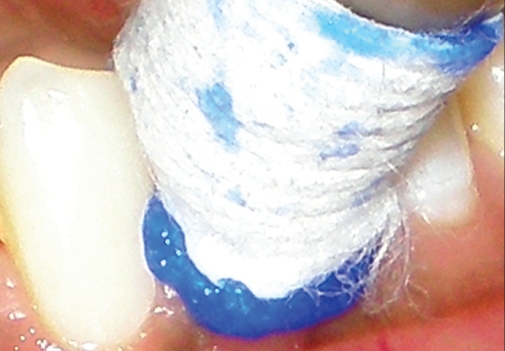
Magic Foam Cord in gingival sulcus of mandibular left 1^st^ premolar

### Retraction procedure for retraction cord

The use of gingival retraction cords with 5% aluminum chloride has been shown to be safe and effective.[7] The labial gingival sulcus of the maxillary left-sided first premolar is rinsed, dried, and isolated with cotton rolls [Figure 1c]. An Ultrapak, 00 #, 5% aluminum chloride impregnated retraction cord is cut for the required length and placed in the sulcus with a cord packer and placed for ten minutes. It is suggested that the placement starts at the interproximal gingival crevice, where there is usually more tissue, and continues circumferentially. After the required period, the time cord was removed, and the gingival sulcus washed and dried.[8]

**Figure 1c:**
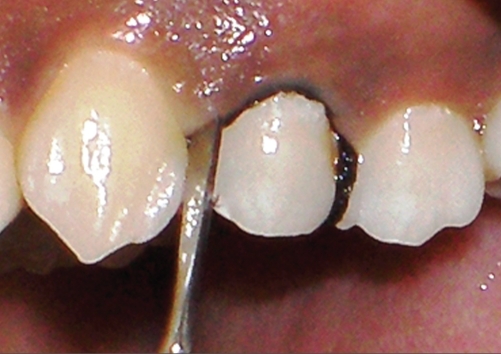
Retraction cord in gingival sulcus of maxillary left 1^st^ premolar

In this case the retraction procedure was performed by the same expert prosthodontist to minimize interexaminer error.

### Extraction and laboratory procedure

As microscopically the features of acute inflammation can be seen as early as within 48 hours, the patients were considered for extractions after 48 hours of retraction. The patient was anesthetized using 2 ml of 2% lidocaine with 1: 50,000 epinephrine. An incision using a No. 15 surgical blade was made facially 2 – 3 mm away from the marginal gingiva. The extraction was performed mainly with an elevator to reduce tissue trauma. The tooth was extracted with the adjacent marginal gingiva, decalcified with 10% formic acid, processed with a series of 70, 80, and 90% absolute alcohol, xylene2, and xylene1. Microscopic sections were obtained by cutting the labiolingual block sections at eight microns, with microtome and staining done with hematoxylin and and eosin stain.[9]

### Histological examination

As the cellular response to the retraction materials was the main interest, the following criteria were used to determine the changes depending upon the relative injury caused by the retraction materials.[9]

Normal – Normal gingival epithelium
Mild – Stripping and desquamation of the epithelium
Moderate – Hydropic degeneration, hyperemia, inflammatory cells
Severe – Epithelial proliferation and necrosis

## RESULT

The histological specimen of the retraction cord revealed that the cord was pressed past the cementoenamel junction with facial displacement of the entire gingival unit. The sulcular epithelium was present, but disrupted. The junctional epithelium was sometimes missing from the outermost border. The residual junctional epithelium displayed intracellular hydropic degeneration, stripping, and desquamation of the epithelium [Figure 2a].

**Figure 2a:**
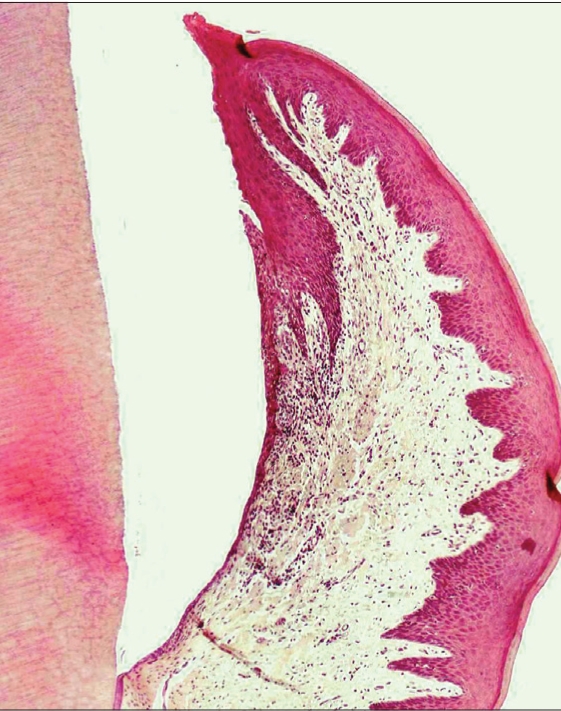
Histologic view with Expasyl

However, the histological specimen of the retraction paste shows only eight cases of disrupted junctional epithelium and sulcular epithelium, as compared to the retraction cord. The remaining specimens show an intact junctional epithelium [Figure 2a and b].

**Figure 2b:**
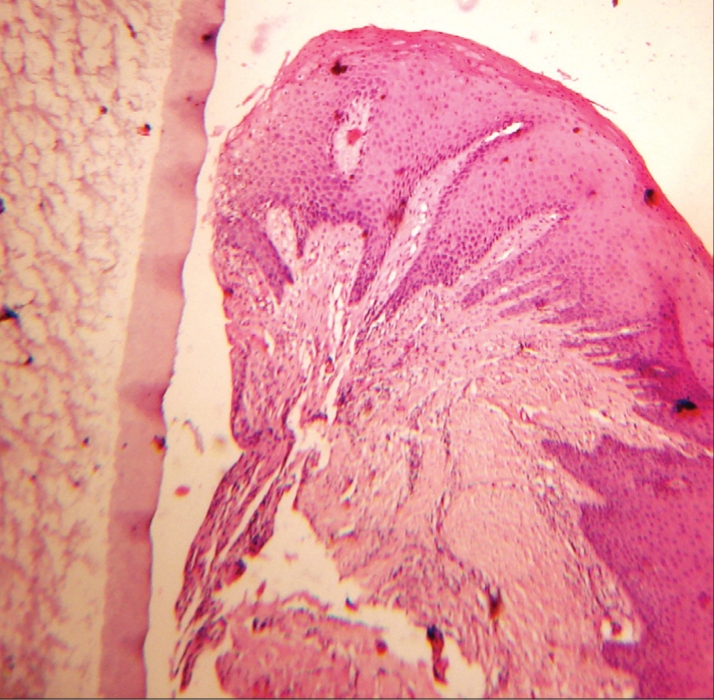
Histologic view with Magic FoamCord

Thus, mechanical and mechanochemical methods do cause injury to the gingival sulcus epithelium, but the injury varies from slight with retraction paste to severe with retraction cord [Figure 2c].

**Figure 2c:**
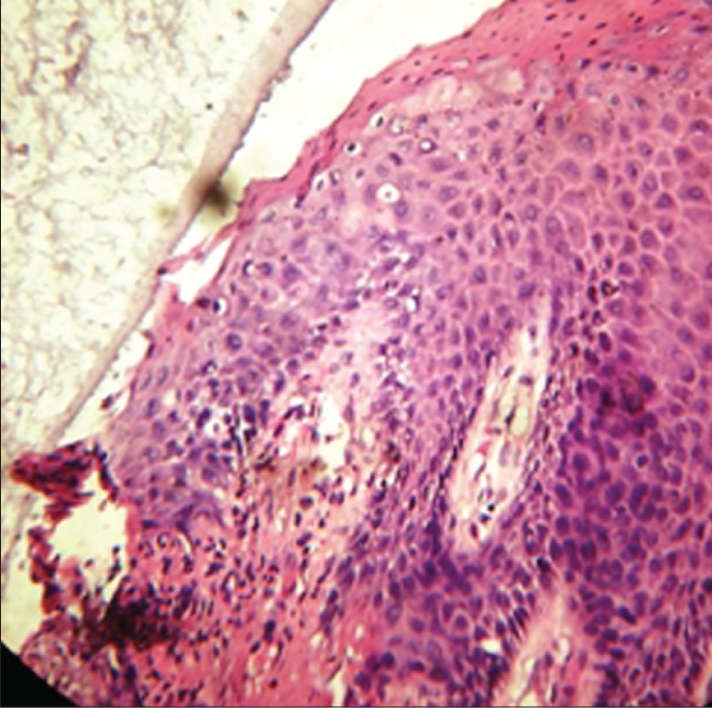
Histologic view with retraction cord

From the observations shown in Table 1, it was perceived that out of the 30 cases studied, mild injury was noticed with the use of Expasyl, Magic Foam Cord, and impregnated retraction cord, of 6.67, 20, and 36.67%, respectively. Moderate injury was observed with the use of impregnated retraction cord in 20% of the cases. No severe injury was observed with the use of different retraction materials.

**Table 1:** Distribution of relative degree of injury caused by the retraction materials

Retraction material	Expasyl with 14 (n=30)	Magic foam cord with 34 (n=30)	Retraction cord with 24 (n=30)
	No. (%)	No. (%)	No. (%)
Normal	28 (93.33)	24 (80)	13 (43.33)
Mild	02 (6.67)	06 (20)	11 (36.67)
Moderate	00	00	06 (20)
Severe	00	00	00

Value of χ^2^ = 23.98, *P*<0.05, Significant, Figures in parenthesis are in percentage

By applying the chi-square test, it has been proved that there is a significant association between different retraction materials and the relative degree of injury to the gingival sulcular epithelium, that is, *P*<0.05.

## DISCUSSION

Although, from periodontal point of view, it is preferable to place the margins of restorations supragingivally, for esthetic or other reasons, the dentist may be forced to place them subgingivally.[10] Other studies using clinical and histopathological evaluation of gingival retraction in humans show that gingival retraction with the cord caused destruction of the junctional epithelium, which took about eight days to heal. The average postoperative gingival recession seen with cord retraction was 0.2±0.1 mm.

The most widely used and popular method is the use of retraction cords. A study by Van der Velden and De Vries has shown that the epithelial attachment sustains injuries at a force of 1 N/mm^2^, while it ruptures at 2.5 N/mm^2^. The cord technique requires almost 2.5 N/mm^2^. The retraction cord achieves the desired retraction, but placing a retraction cord is not an easy method.[6] It needs physical manipulation of the tissue, leading to gingival bleeding. Thus, use of a retraction cord has the risk of epithelial attachment injury, pain during cord placement, sometimes requiring local anesthesia. Also, more time is required, and it may initiate gingival bleeding and oozing.

A complete paradigm shift has been made with the introduction of a very novel idea to achieve retraction and hemostasis at the same time. In our study we compared the two retraction materials: Expasyl and Magic Foam Cord with the conventional retraction cord. We used the maxillary right first premolar for gingival retraction with Expasyl and the mandibular left first premolar with Magic Foam Cord. The fundamental principle of the Expasyl was to insert a stiff, hemostatic, plastic, non-setting material into the gingival sulcus under mild pressure and allow the material to stay in place for 1 – 2 min6. In our study, the histological specimen of the retraction cord revealed that the disrupted sulcular epithelium and junctional epithelium were sometimes missing. Also, the junctional epithelium displayed intracellular hydropic degeneration, stripping, and desquamation of epithelium. These findings are similar to Jon Ruel *et al*.[8] and R. Azzi *et al*.[10] The histological specimens of the retraction paste showed only six cases of disrupted junctional epithelium and sulcular epithelium as compared to the retraction cord. The remaining specimens showed an intact junctional epithelium. According to Patrick Lesage and Mona Kakar, the material under pressure caused sufficient displacement of the gingival tissue and this displacement stayed in place long enough for either recording of the impression or to carry out the restorative or bonding procedures.[6] It was noninvasive, simple to use, painless, reliable, a hemostatic agent, effective, safe, increased patient comfort, and saved time.

Magic Foam Cord is a product for an easy, nontraumatic, and less time consuming retraction of the sulcus. It is biologically very compatible, with no adverse side effects or interactions. Polyvinylsiloxane has a high tear resistance. The technique is faster and easier than the use of retraction cords or scalpel / rotary instruments.

## CONCLUSION

To conclude, the results of the present study clearly reveal that there is a significant association between retraction materials and the gingival sulcular epithelium. It can be stated that the impregnated retraction cord, may be used frequently, but it needs proper tissue manipulation and is technique sensitive.

A definite alternative for gingival retraction now exists in the form of retraction paste (Expasyl / Magic Foam Cord). In regard to hemostasis, there is no doubt about the efficacy of these materials and their ability to be extremely effective clinically. The retraction procedure also appears very safe and easy to use. Thus, the newly advanced material in the form of retraction pastes like Expasyl or Magic Foam Cord have been found to be better than the cord, as assessed histologically, with respect to the periodontium. The patient tolerance was observed to be very good. No anesthesia was required and the material exhibited total biocompatibility.

### Future research

The long-range effects of the marginal fit are probably the most important factors for enhancing periodontal health. This study has involved only healthy periodontal subjects. Different effects on the junctional epithelium may be observed in tissues, characterized by gingivitis or periodontitis. A broader study involving a greater range of procedures and conditions is recommended, to evaluate each retraction technique. This study has involved teeth that have an adequate zone of attached gingiva. More complicated and perhaps altered sequences may be observed if the procedures are performed on gingival margins of alveolar mucosa, thin gingival walls or areas of root prominence and thin cortical bone.
